# Follicle-Stimulating Hormone Receptor (FSHR) Ser680Asn Genotype Does Not Affect the Follicular Fluid Hormonal Profile in Stimulated Cycles Using Different Gonadotropin Preparations for Ovulation Induction: A Pilot Study

**DOI:** 10.7759/cureus.62116

**Published:** 2024-06-10

**Authors:** Myrto-Sotiria Papamentzelopoulou, Despoina Mavrogianni, Emmanouela Liokari, Sofoklis Stavros, Anastasios Potiris, Dimitris Doumplis, Dimitrios Loutradis

**Affiliations:** 1 1st Department of Obstetrics and Gynecology, Alexandra General Hospital, National and Kapodistrian University of Athens School of Medicine, Athens, GRC; 2 Department of Obstetrics and Gynecology, Fertility Institute, Athens, GRC; 3 3rd Department of Obstetrics and Gynecology, Attikon University Hospital, National and Kapodistrian University of Athens, Athens, GRC; 4 Department of Obstetrics and Gynecology, National and Kapodistrian University of Athens, Athens, GRC

**Keywords:** fshr ser680asn genotype, pregnancy rate, embryo quality, maturation rate, ovulation induction, hormonal profile, follicular fluid

## Abstract

Background: The existing literature lacks consensus on the effectiveness of utilizing polymorphisms to enhance outcomes in in vitro fertilization (IVF), particularly regarding ovulation induction protocols, oocyte and embryo quality, and pregnancy rates. Therefore, the present pilot study aims to assess whether the composition of different gonadotropin preparations affects the ovarian stimulation protocol concerning follicle-stimulating hormone receptor (*FSHR*) Ser680Asn genotypes (Ser/Ser, Ser/Asn, and Asn/Asn), in terms of ovulation induction parameters, including oocyte maturation rate, embryo quality, and pregnancy rate.

Methodology: A total of 94 IVF patients underwent treatment using a GnRH antagonist protocol with four distinct gonadotropin preparations: HMG, HMG/hCG, rFSH, and rFSH/hCG. Follicular fluid (FF) samples were pooled for each patient for analysis.

Results: No statistical differences in the FF hormonal profile (progesterone, testosterone, androstenedione, estradiol, FSH, hCG) among the *FSHR* genotypes were reported either separately for each protocol or in combination for the four different preparations of gonadotropins. The maturation rate of MII oocytes and embryo quality did not differ among women carrying either Ser/Ser, Ser/Asn, or Asn/Asn genotype (p-value=0.475, and p-value=1.000, respectively). Moreover, no statistically significant correlation was revealed among Ser/Ser, Ser/Asn, and Asn/Asn carriers and pregnancy rate (p = 0.588).

Conclusions: FF hormonal analysis of women undergoing IVF using different ovulation induction protocols and carrying either Ser/Ser, Ser/Asn, or Asn/Asn genotype revealed no significant correlations, in terms of maturation rate of MII oocytes, embryo quality, and pregnancy rate, indicating that the *FSHR *Ser680Asn genotype does not constitute a biomarker for a positive pregnancy outcome. Therefore, the existence of a different mechanism for the expression of *FSHR* Ser680Asn genotypes in the FF hormonal profile related to stimulated cycles is implied.

## Introduction

The ovarian response to follicle-stimulating hormone (FSH) action varies significantly among women and factors possibly affecting such response are investigated. Therefore, researchers identify the genes that are involved in the response to FSH stimulation by applying pharmacogenetics to assisted reproduction techniques (ARTs) [1].

Interestingly, exploring the molecular events inside the follicular fluid (FF) during ovulation induction and in the natural FF could be highly informative for identifying genetic markers serving as potential predictive tests prior to ovarian stimulation for the required FSH dose determination, the management of related to FSH stimulation complications, and the ovarian response prediction. Recently, insights have been gained in identifying and characterizing variants in the gene that encodes the FSH receptor (FSHR) [2]. Several polymorphisms of the *FSHR *gene have been identified and have gained attention, including Ser680Asn, Thr307Ala, -29, and FSHβ -211G>T [3], due to the differential response to exogenous FSH administration, potentially modifying the outcome of ovarian stimulation [4]. Accordingly, the investigation of such *FSHR *gene variants should be considered in controlled ovarian hyperstimulation during assisted reproduction in women with normal ovarian function to gather valuable information about how each patient reacts to exogenous gonadotrophin administration during ovulation induction [5].

Currently, possible associations between the polymorphisms at position 680 of the *FSHR *gene and the ART outcomes have been explored. In particular, this variation constitutes one of the two substitutions of exon 10 (rs6166) [2,6]. It results in the change of the nucleotide guanine to adenine (c.2039 G>A), which leads to the replacement of serine with asparagine at position 680 (Asn680Ser) [6,7]. Several studies indicate significant diversity in the response to controlled ovarian stimulation (COS) following in vitro fertilization (IVF)/intracytoplasmic sperm injection (ICSI) protocols based on Ser680Asn genotypes (G/G-Ser/Ser, G/A-Ser/Asn, A/A-Asn/Asn) per se or combined with polygenic analysis of various (Estrogen Receptor 1) *ESR1 *and (Estrogen Receptor 2) *ESR2 *gene polymorphisms [2,4,8].

The first *FSHR *SNP studied was the Ser680Asn [9-11]. In 2000, Mayorga et al. investigated the role of FSH receptor variants in relation to exogenous administered FSH in women undergoing ovarian stimulation during IVF [11]. As disclosed therein, women homozygous for the Ser allele (Ser/Ser) required higher doses of exogenous administered FSH compared to women carrying the Asn allele (Asn/Asn and Ser/Asn). Serum FSH levels were higher in women with the Ser/Ser genotype and lower in women with the Asn/Asn genotype. Loutradis et al. reported that Greek patients more often carry the Ser/Asn genotype, correlated with more follicles and oocytes in both poor and good responder patients, while the Ser/Ser variant was associated with higher serum FSH levels, and the Ser/Asn with lower levels [12]. Yao et al. and Greb et al. disclosed similar results to those reported by Mayorga et al. [13,14]. In 2003, de Castro et al. observed a higher proportion of IVF cycle cancellations in women carrying the Ser/Ser genotype, a greater distribution of this genotype in poor responders, and a lower response rate to stimulation to recombinant FSH [15]. In contrast, as demonstrated in the study of Klinkert et al., the implantation and pregnancy rates were higher in women carrying the Ser/Ser genotype [16], while other controversies were also reported [3,17].

Various protocols have evaluated the use of different forms of gonadotropins, either of human purified urine or artificial recombinant origin [18], with urine analogues including HMG, urinary FSH (uFSH), and urinary hCG (uhCG) and recombinant regimens including recombinant FSH (rFSH), recombinant LH (rLH), and recombinant hCG (rhCG). The challenge remains the selection of the most appropriate gonadotropin regimen between uFSH and rFSH, with no significant differences being currently reported in terms of the number of oocytes retrieved or pregnancy rates between the two types of FSH [19,20]. Hence, no consensus is available on optimal regimens and preparations [21]. A recent meta-analysis investigated whether separate genotypes of *FSHR *Ser680Asn could influence the outcome of COS in patients following IVF/ICSI protocols. As disclosed, women carrying the Asn/Asn genotype presented elevated estradiol (E2) on the day of human chorionic gonadotropin (hCG) administration, but fewer embryos for transfer compared to women carrying the Ser/Ser genotype. Higher E2 levels on the day of hCG administration were also reported in women carrying the Ser/Asn genotype [4].

Several studies have been conducted to examine the hormonal profile of the FF derived from different protocols of ovulation induction to correlate these parameters with the clinical data of IVF procedures, such as the maturation rate of oocytes, embryo quality, and pregnancy rate [21-24].

Borgbo et al. observed that in follicles >6 mm and in the presence of the Asn/Asn genotype in the *FSHR *307/680 polymorphisms, E2 levels were significantly higher in human small antral follicles (hSAF) collected under physiological FSH conditions [25]. Also, the impacts of the *FSHR *−29G>A genotype on the hormone profile in FF from hSAF were examined, where the androgen levels of hSAF were significantly elevated in the Ans/Asn and Ser/Asn genotypes in the FSHR promoter polymorphism *FSHR *−29G>A [26].

With this notable data, the present study was designed for the first time to examine the relationship of *FSHR *Ser680Asn genotypes (Ser/Ser, Ser/Asn, and Asn/Asn) with the hormone profile of progesterone, testosterone, androstenedione, estradiol, FSH, and hCG in the FF derived from four different protocols of multiple ovulation induction. The goal is to examine the hormonal profile of the FF in stimulated cycles with different gonadotropin preparations.

## Materials and methods

Study population

The present pilot study was conducted from January 2022 to October 2022 at Fertility Institute S.A., Athens, Greece. The study group comprised 94 women who underwent COS in a GnRH antagonist protocol with an age range of 24-45 years. Patient recruitment was accomplished using a computer-generated randomization table. Four different regimens were used: HMG (n=21) (Menopour Ferring), HMG/hCG (n=23), rFSH (n=29) (Gonal-F Merck), and rFSH/hCG (n=21). The addition of hCG (Pregnyl MSD) was a low dose of 100 IU/day. Protocol selection was based on age, anti-Müllerian hormone (AMH), FSH, luteinizing hormone (LH), and antral follicle count (AFC). When the age of the patient was lower than 35 years, rFSH was administered, while when the age was greater than 35 years, HMG, rFSH+hCG, or HMG+hCG were administered. The rationale for this decision was that women of advanced age are likely to achieve pregnancy using LH or hCG activity.

Inclusion criteria constituted women presenting no uterine or ovarian anomalies, both ovaries intact, a normal hormonal profile according to WHO guidelines, and a regular menstrual cycle of 25-30 days. The indications for fertility treatment included male factor, tubal factor, and unexplained infertility. None of the participants had undergone ovarian stimulation or any other hormonal treatment for at least three months before entering the ART protocol. Demographic and clinical data, such as age and BMI, and early follicular phase FSH, LH, prolactin (PRL), AMH, thyroid-stimulating hormone (TSH), T3, T4, TPO, and TG levels within the preceding six months were recorded. In addition, the number of follicles, mature oocytes and embryos, and the subsequent pregnancy rates were recorded for each study participant.

Embryo quality evaluation was blinded according to treatment and included the assessment of blastomere number, degree of fragmentation, blastomere uniformity, and multinucleation [27]. Embryo transfer was performed on day 5 after oocyte retrieval. The main outcome measures were intrafollicular hormone concentrations, including progesterone, testosterone, androstenedione, estradiol, FSH, and hCG, in relation to Asn680Ser genotypes: Ser/Ser, Ser/Asn, and Asn/Asn. The study protocol was approved by the review board of the Fertility Institute. All participants provided informed consent for their medical records to be used in the study.

Ovarian stimulation, IVF/ICSI, and embryo transfer

GnRH-Antagonist Protocol

The study participants underwent COS in a GnRH antagonist protocol according to the strict routine practice of the institute. A baseline ultrasound scan was performed on cycle day 2. In the case of not indicative scan findings, serum estradiol and progesterone levels were determined. On cycle day 5, daily administration of GnRH-antagonist (Orgalutran, MSD, Hellas) was initiated and maintained until triggering of final oocyte maturation with rhCG (Ovitrell, Merck Hellas). On day 3, gonadotropin administration was initiated at a dose of 200 IU, which was adjusted according to ovarian response on a daily basis, six days after the onset of gonadotropin administration. On day 2 of the cycle and throughout the follicular phase, hCG (Pregnyl, MSD, Hellas) was administered intramuscularly at a dose of 100 IU per day along with gonadotropins, until the day of final oocyte maturation triggering.

Serum E2 levels were measured daily from day 5 of ovarian stimulation with gonadotropins until the day of final oocyte maturation triggering by subcutaneous administration of 250 μg rhCG (Ovitrell, Merck, Hellas). Follicular tracking started on day 6 of stimulation and subsequent ultrasound scans were performed daily until oocyte retrieval. Follicular aspiration and oocyte retrieval followed 36h after rhCG administration via transvaginal ultrasound-guided puncture. The luteal phase was supported with 200 mg of micronized progesterone administered intravaginally three times daily starting from the day after egg collection onward and serum β-hCG was measured 14 days later. Clinical pregnancy was defined as the presence of a gestational sac on ultrasound at seven gestational weeks. Hormonal profile measurements were performed in the institute. The fertility specialists of the institute conducted ultrasound scans, oocyte retrievals, and embryo transfers, while the two senior embryologists of the institute performed oocyte grading, fertilization, early embryo development, and embryo grading.

FF hormonal measurements

Oocyte retrieval took place 36 hours after rhCG triggering. The procedure was performed using a needle single lumen (Cook Medical, USA) with manual aspiration of each single follicle. FFs from follicles of approximately 12 mm and greater were aspirated and centrifuged, and supernatants were aliquoted and stored at -20°C for analysis. Subsequent hormonal measurements in FFs, including progesterone (Prg), testosterone (T), androstenedione (A), hCG, FSH, and estradiol (Ε2), were performed at the Laboratory Genes Lab (Athens, Greece). Estradiol and progesterone required a 1:1000 dilution. The RIA method was applied for androstenedione determination. For all other hormones, COBAS 6000 analyzer (COBAS 6000; Roche Diagnostics) was used. The sensitivity for each measurement was as follows: hCG 0.1 IU/L; FSH, 0.1 IU/L; LH 0.1 IU/L; E2 0.02 nmol/L; progesterone 0.1 nmol/L; T 0.087 nmol/L; and androstenedione, 0.1 nmol/L.

Genotyping

Peripheral blood was collected from study participants to perform *FSHR *Ser680Asn genotyping analysis. The samples were stored at -20°C. DNA isolation was conducted using the PureLink Genomic DNA kit (Invitrogen, USA), following the manufacturer’s instructions. Real-time polymerase chain reaction (RT-PCR) was applied for the detection of Ser680Asn polymorphism, using the LightCycler 480II (RocheGmBH Manheim, Germany). The sequences of the FSHR-specific primers and probes used were as follows: FSHR S AGTGTGGCTGCTATGAAATGC, FSHR A GGCTAAATGACTTAGAGGGACAAGTA, SP CCCAGAGTCACCAATGGTXITCCA-PH.

Statistical analysis

The qualitative data were presented as frequency and percentage. Associations were explored by the Fisher’s exact test between protocols and *FSHR *genotype categorical variables. The quantitative data were presented as mean and standard deviation (or 95% confidence interval for mean). Statistical analysis was performed using IBM SPSS Statistics for Windows, Version 20 (Released 2011; IBM Corp., Armonk, New York, United States). The criterion of statistical significance was set at 5%.

## Results

The study group consisted of 94 women with a mean age of 36.3 ± 4.5 years (min=24, max=45). Of them, 21 (22.3%) were treated with HMG, 23 (24.5%) with HMG/hCG, 29 (30.9%) with rFSH, and 21 (22.3%) with rFSH/hCG. Demographic and clinical characteristics, including age, years of infertility, previous attempts, BMI, and hormonal profile (FSH, LH, PRL, AMH, TSH, T3, T4, FT3, FT4, aTPO, aTG), are presented in Table 1, revealing no statistically significant differences among the Ser/Ser, Ser/Asn, and Asn/Asn genotypes of the *FSHR *gene. It should be clarified that some data is more spread out and presents a higher degree of variability leading to greater SD values compared to mean values.

**Table 1:** Demographic and clinical characteristics in relation to the FSHR Ser680Asn genotypes. The data has been represented as mean±SD and p<0.05 is considered significant. FSH: Follicle-stimulating hormone; AMH: anti-Müllerian hormone; TSH: thyroid-stimulating hormone; PRL: prolactin; LH: luteinizing hormone

Demographical and Clinical Characteristics	Ser/Ser	Ser/Asn	Asn/Asn	p-value
Age	36.15±3.75	35.91±5.03	38.33±3.63	0.248
Years of infertility	3.94±3.55	4.25±3.60	5.83±4.9	0.455
Number of previous attempts	0.76±1.17	1.11±1.54	0.67±0.89	0.522
BMI	23.12±3.66	23.81±4.36	24.87±7.40	0.790
FSH (IU/L)	7.30± 3.55	7.86± 2.71	7.60±2.11	0.569
LH (IU/L)	7.11± 3.63	6.89 ± 3.02	5.95 ± 2.25	0.731
PRL (ng/ml)	17.57 ± 9.72	16.48 ± 10.54	16.97±10.65	0.669
AMH (ng/ml)	3.13±2.03	4.40±8.93	2.18±1.07	0.404
TSH (ng/ml)	1.96±0.76	2.10±1.09	1.65±0.70	0.223
T3 (ng/ml)	14.59 ± 46.42	9.00 ± 33.19	12.6 ± 31.02	0.315
T4 (μg/dl)	21.15 ± 29.93	75.87 ± 273.51	8.02 ± 0.82	0.539
FT3 (ng/dl)	3.64± 0.85	3.38 ± 0.91	2.55±1.13	0.155
FT4 (pg/ml)	4.95 ± 5.98	3.78 ± 5.75	3.04 ± 5.25	0.641
a-TΡO (IU/ml)	638.04 ± 2116.02	109.30 ± 328.17	67.98± 124.81	0.853
a-TG (IU/ml)	104.63 ± 188.07	53.98± 92.31	47.61± 54.10	0.574
Values are mean ± SD One-way ANOVA or Kruskal-Wallis test or Fisher's exact test is used				

Table 2 presents the characteristics of ovulation induction in relation to *FSHR *Ser680Asn genotypes. As observed, the consumption of gonadotropins, the days of ovulation, the E2 level on hCG administration, the number of collected oocytes, and the number of embryos were similar within all *FSHR *genotypes.

**Table 2:** Ovulation induction characteristics in relation to the FSHR Ser680Asn genotypes. The data has been represented as mean±SD and p<0.05 is considered significant.

Ovulation Induction Characteristics	Ser/Ser	Ser/Asn	Asn/Asn	p-value
Consumption of gonadotropins IU	3946.97 ± 1069.14	4172.16 ± 1861.02	3764.58 ± 1211.66	0.605
Days of ovulation induction	9.36±1.29	9.95±1.57	9.25±1.66	0.101
E2 on day of hCG administration	2526.24±2258.21	2274.02±1129.38	2858.08±1604.26	0.536
Number of follicles > 12mm	9.58 ± 2.2.62	9.98 ± 3.27	10.17 ± 2.72	0.874
Number of oocytes	8.88 ± 2.51	9.32 ± 3.33	9.17 ± 2.82	0.882
Number of embryos	6.78 ± 2.11	7.43 ± 3.27	7.58 ± 2.61	0.649
Values are mean ± SD				
One-way ANOVA and Fisher's exact test are used				

Moreover, statistical analysis of the FF hormonal profile in Ser/Ser, Ser/Asn, and Asn/Asn polymorphisms conducted separately for each protocol and in combination for the four different preparations of gonadotropins did not find a statistical difference in hormone profiles (Prog, Test, Andr, E2, FSH, hCG) among the *FSHR* genotypes (Table 3).

**Table 3:** Follicular fluid hormonal profile in relation to the FSHR Ser680Asn genotypes. The data has been represented as mean±SD and p<0.05 is considered significant. FSH: Follicle-stimulating hormone

Follicular Fluid Hormonal Profile	Ser/Ser	Ser/Asn	Asn/Asn	p-value
Prg (ng/ml)	22068 ± 11040	19444 ± 12181	19877 ± 10933	0.425
T (ng/ml)	416.07 ± 414.89	410.79± 359.76	493.51± 417.36	0.789
A (ng/ml)	5.47 ± 2.802	5.34 ± 2.73	5.02± 2.95	0.959
hCG (mIU/ml)	58.53 ± 40.27	49.21± 30.77	57.19 ± 28.75	0.354
FSH (IU/L)	6.87 ± 2.90	6.89± 3.31	7.09± 2.85	0.841
E2 (ng/ml)	25965388 ± 138963560	3460570 ± 12190481	9242658± 26498577	0.714
Values are mean ± SD				
One-way ANOVA and Fisher's exact test are used				

Regarding the maturation rate of MII oocytes, there was no statistically significant correlation between the *FSHR *genotypes (Ser/Ser and Ser/Asn versus Asn/Asn) and the number of mature oocytes (MII) (p-value=0.475, Table 4).

**Table 4:** Maturation rate of MII oocytes in relation to the FSHR Ser680Asn genotypes. The data has been represented as mean±SD and p<0.05 is considered significant.

MII mature oocytes	n	Mean±SD		p-value
Ser/Ser + Ser/Asn	79	7.99	2.82	0.475
Asn/Asn	14	8.58	2.94	

Furthermore, the statistical analysis of the morphological quality of embryos (good-quality embryos and poor-quality embryos) did not differ significantly among patients who carried either Ser/Ser, Ser/Asn or Asn/Asn genotype (p-value=1.000, Table 5).

**Table 5:** Quality of embryos in relation to the FSHR Ser680Asn genotypes. The data has been represented as N and p<0.05 is considered significant.

*FSHR *genotypes	Embryo morphological quality			
	Good-quality embryos	Poor-quality embryos	Total	p-value
Ser/Ser+Ser/Asn	73	3	76 (100%)	
Asn/Asn	14	0	14 (100%)	
Total	87	3	90 (100%)	1.000
Values are numbers (percentages) and Fisher's exact test is used				

Ultimately, potential association between *FSHR* genotypes and pregnancy rate was investigated. As presented in Table 6, no statistically significant correlation was revealed between Ser/Ser, Ser/Asn, and Asn/Asn carriers and pregnancy rate (p = 0.588), indicating that the *FSHR *Ser680Asn genotype does not constitute a biomarker for a positive pregnancy outcome.

**Table 6:** Pregnancy rate in relation to the FSHR Ser680Asn genotypes. The data has been represented as N and % and p<0.05 is considered significant.

			Pregnancy		Total
			No	Yes	
*FSHR *genotype	Ser/Ser	Count	23	10	33
		%	69.7%	30.3%	100.0%
	Ser/Asn	Count	35	9	44
		%	79.5%	20.5%	100.0%
	Asn/Asn	Count	10	2	12
		%	83.3%	16.7%	100.0%
Total		Count	68	21	89
		%	76.4%	23.6%	100.0%
				p-value	0.588
Values are numbers (percentages) and Fisher's exact test is used					

## Discussion

The present pilot study aimed at investigating the impact of the *FSHR *Ser680Asn genotypes (Ser/Ser, Ser/Asn, and Asn/Asn) in relation to the endocrine profile of FFs, including progesterone, estradiol, testosterone, FSH, hCG, and androstenedione, generated after gonadotrophin stimulation. Different regimens (HMG, HMG/hCG, rFSH, and rFSH/hCG) within a GnRH antagonist protocol were utilized.

As demonstrated, ovulation induction parameters, including the number of gonadotropins, days of ovulation induction, and E2 levels on the day of hCG administration, were similar among women carrying either Ser/Ser, Ser/Asn, or Asn/Asn genotype. Moreover, *FSHR *gene genotypes did not seem to affect the FF hormonal profiling (progesterone, testosterone, androstenedione, FSH, hCG, estradiol) for both each protocol separately and the combination of all protocols.

Regarding the examined intrafollicular hormone concentrations in normal small antral follicles with *FSHR *307/680 polymorphisms, a significant change was observed between *FSHR *307/680 polymorphisms in human small antral follicles retrieved under physiological conditions. Estradiol levels were significantly higher for the Ser/Ser genotype in follicles >6 mm, while progesterone, testosterone, and androstenedione did not exhibit statistically significant differences between the *FSHR* genotypes [25]. They suggested that follicle selection takes place around this diameter, and the selected follicle responds to gonadotrophin levels by increasing its production of estradiol. It should be noted that in this study, human antral follicles were collected on various days during the menstrual cycle. Since FSH levels vary across the menstrual cycle, the intrafollicular hormone milieu may not accurately represent the actual FF milieu. In our study where the derived FF was from stimulated cycles with different gonadotropin preparations, we did not observe any differences in estradiol or androgen levels based on carriers’ genotypic profile. A possible explanation could be that the follicles were collected in natural cycles on different days of the menstrual cycle where FSH expression varied depending on the day of the cycle.

Studies by Borgbo et al. examined the *FSHR *polymorphisms in two different loci (370/680 and -29) using the FF from almost 200 follicles collected on different days of the menstrual cycle [25,26]. Differences observed reflect the influence of *FSHR *genotypes on intrafollicular conditions, such as higher E2 levels in the *FSHR *680 Asn/Asn genotype when the follicle diameter was >6mm, while androgen levels did not show any difference regardless of the *FSHR *genotypes. On the other hand, when they examined the -29 *FSHR *locus, they showed an increased level of androgen in Asn/Asn and Ser/Asn genotypes. This observation shows that in the same gene but in a different locus, the polymorphism is expressed differently depending on the location of the polymorphism. Regarding the present study at the 680 loci, the hormonal profile was similar in the three *FSHR *genotypes, where the follicles derived from different ovulation induction gonadotropins. The hormonal profile (Prog, Test, Andr, E2, FSH, hCG) did not differentiate between the different gonadotropin preparations in all *FSHR *genotypes, indicating that there should be a different mechanism in stimulated and unstimulated follicles.

The maturation rate of MII oocytes and quality of embryos were not affected by the *FSHR *Ser680Asn genotypes, a finding that is in alignment with the metanalysis by Prodromidou et al. [4]. Moreover, in our dataset, 21 (23.6%) women became pregnant, with 19 carrying either the Ser/Ser or the Ser/Asn genotype, and only two carrying the Asn/Asn genotype, suggesting a tendency of a favorable pregnancy outcome in the presence of the Ser allele; however, such observation did not reach statistical significance. In the above-mentioned meta-analysis [4], it was shown that the Ser/Ser genotype was associated with a higher number of transferable embryos and an enhanced pregnancy rate; thus, suggesting Ser allele as a potential marker of ovarian response to COS.

It is worth considering whether supraphysiological conditions in stimulated cycles affect the function of SNPs differently than in natural cycles regarding the hormonal profile of the FF. In our study, the hormonal profile of progesterone, testosterone, androstenedione, hCG, FSH, and estradiol in the FF from women undergoing IVF using different ovulation induction protocols revealed no correlation between FF hormonal profiles and *FSHR *Ser680Asn genotypes.

Nevertheless, the limitations of the present pilot study are the small sample size and caution should be taken upon results interpretation. Further studies with larger study populations and multiple research groups are needed to draw more meaningful and reliable conclusions about the role of hormones in the FF in stimulated cycles and the possible effects of *FSHR *genotypes in 680 loci. This collaborative and iterative approach strengthens the scientific understanding of the topic and increases the credibility of the results.

## Conclusions

The FF hormonal profile from women undergoing IVF using different ovulation induction protocols and carrying either Ser/Ser, Ser/Asn, or Asn/Asn genotype revealed no significant correlations, in terms of ovulation induction parameters, including maturation rate of MII oocytes, embryo quality, and pregnancy rate. Ultimately, the present pilot study suggests that there is no strong evidence that a specific *FSHR *genotype correlates with a favorable IFV outcome; thus, it cannot be used solely as an effective tool to predict the IVF outcome. Therefore, future large-scale studies are needed to unveil the existence of a different mechanism for the expression of *FSHR *Ser680Asn genotypes in the FF hormonal profile in stimulated cycles.

## References

[1] Loutradis D, Vlismas A, Drakakis P, Antsaklis A (2008). Pharmacogenetics in ovarian stimulation--current concepts. Ann N Y Acad Sci.

[2] Pabalan N, Trevisan CM, Peluso C, Jarjanazi H, Christofolini DM, Barbosa CP, Bianco B (2014). Evaluating influence of the genotypes in the follicle-stimulating hormone receptor (FSHR) Ser680Asn (rs6166) polymorphism on poor and hyper-responders to ovarian stimulation: a meta-analysis. J Ovarian Res.

[3] Altmäe S, Hovatta O, Stavreus-Evers A, Salumets A (2011). Genetic predictors of controlled ovarian hyperstimulation: where do we stand today?. Hum Reprod Update.

[4] Prodromidou A, Dimitroulia E, Mavrogianni D, Kathopoulis N, Pappa KI, Loutradis D (2023). The effect of the allelics of Ser680Asn polymorphisms of follicle-stimulating hormone receptor gene in IVF/ICSI cycles: a systematic review and meta-analysis. Reprod Sci.

[5] Gromoll J, Simoni M (2005). Genetic complexity of FSH receptor function. Trends Endocrinol Metab.

[6] Mohiyiddeen L, Nardo LG (2010). Single-nucleotide polymorphisms in the FSH receptor gene and ovarian performance: future role in IVF. Hum Fertil (Camb).

[7] Simoni M, Gromoll J, Hoppner W, Kamischke A, Krafft T, Stähle D, Nieschlag E (1999). Mutational analysis of the follicle-stimulating hormone (FSH) receptor in normal and infertile men: identification and characterization of two discrete FSH receptor isoforms. J Clin Endocrinol Metab.

[8] Anagnostou E, Mavrogianni D, Theofanakis C (2012). ESR1, ESR2 and FSH receptor gene polymorphisms in combination: a useful genetic tool for the prediction of poor responders. Curr Pharm Biotechnol.

[9] Sudo S, Kudo M, Wada S, Sato O, Hsueh AJ, Fujimoto S (2002). Genetic and functional analyses of polymorphisms in the human FSH receptor gene. Mol Hum Reprod.

[10] Simoni M, Casarini L (2014). Mechanisms in endocrinology: genetics of FSH action: a 2014-and-beyond view. Eur J Endocrinol.

[11] Mayorga MP, Gromoll J, Behre HM, Gassner C, Nieschlag E, Simoni M (2000). Ovarian response to follicle-stimulating hormone (FSH) stimulation depends on the FSH receptor genotype. J Clin Endocrinol Metab.

[12] Loutradis D, Patsoula E, Minas V, Koussidis GA, Antsaklis A, Michalas S, Makrigiannakis A (2006). FSH receptor gene polymorphisms have a role for different ovarian response to stimulation in patients entering IVF/ICSI-ET programs. J Assist Reprod Genet.

[13] Yao Y, Ma CH, Tang HL, Hu YF (2011). Influence of follicle-stimulating hormone receptor (FSHR) Ser680Asn polymorphism on ovarian function and in-vitro fertilization outcome: a meta-analysis. Mol Genet Metab.

[14] Greb RR, Behre HM, Simoni M (2005). Pharmacogenetics in ovarian stimulation - current concepts and future options. Reprod Biomed Online.

[15] de Castro F, Ruiz R, Montoro L (2003). Role of follicle-stimulating hormone receptor Ser680Asn polymorphism in the efficacy of follicle-stimulating hormone. Fertil Steril.

[16] Klinkert ER, te Velde ER, Weima S (2006). FSH receptor genotype is associated with pregnancy but not with ovarian response in IVF. Reprod Biomed Online.

[17] Casarini L, Pignatti E, Simoni M (2011). Effects of polymorphisms in gonadotropin and gonadotropin receptor genes on reproductive function. Rev Endocr Metab Disord.

[18] Casarini L, Brigante G, Simoni M, Santi D (2016). Clinical applications of gonadotropins in the female: assisted reproduction and beyond. Prog Mol Biol Transl Sci.

[19] van Wely M, Kwan I, Burt AL, Thomas J, Vail A, Van der Veen F, Al-Inany HG (2011). Recombinant versus urinary gonadotrophin for ovarian stimulation in assisted reproductive technology cycles. Cochrane Database Syst Rev.

[20] Thuesen LL, Andersen AN, Loft A, Smitz J (2014). Intrafollicular endocrine milieu after addition of hCG to recombinant FSH during controlled ovarian stimulation for in vitro fertilization. J Clin Endocrinol Metab.

[21] Smitz J, Andersen AN, Devroey P, Arce JC (2007). Endocrine profile in serum and follicular fluid differs after ovarian stimulation with HP-hMG or recombinant FSH in IVF patients. Hum Reprod.

[22] Hillier SG, De Zwart FA (1981). Evidence that granulosa cell aromatase induction/activation by follicle-stimulating hormone is an androgen receptor-regulated process in-vitro. Endocrinology.

[23] Basuray R, Rawlins RG, Radwanska E (1988). High progesterone/estradiol ratio in follicular fluid at oocyte aspiration for in vitro fertilization as a predictor of possible pregnancy. Fertil Steril.

[24] Nordhoff V, Sonntag B, von Tils D (2011). Effects of the FSH receptor gene polymorphism p.N680S on cAMP and steroid production in cultured primary human granulosa cells. Reprod Biomed Online.

[25] Borgbo T, Jeppesen JV, Lindgren I, Lundberg Giwercman Y, Hansen LL, Yding Andersen C (2015). Effect of the FSH receptor single nucleotide polymorphisms (FSHR 307/680) on the follicular fluid hormone profile and the granulosa cell gene expression in human small antral follicles. Mol Hum Reprod.

[26] Borgbo T, Klučková H, Macek M Sr, Chrudimska J, Kristensen SG, Hansen LL, Andersen CY (2017). The common follicle-stimulating hormone receptor (FSHR) promoter polymorphism FSHR -29G >  A affects androgen production in normal human small antral follicles. Front Endocrinol (Lausanne).

[27] Loutradis D, Drakakis P, Kallianidis K, Milingos S, Dendrinos S, Michalas S (1999). Oocyte morphology correlates with embryo quality and pregnancy rate after intracytoplasmic sperm injection. Fertil Steril.

